# Reduction of Energy Intensity in Broiler Facilities: Methodology and Strategies

**DOI:** 10.3389/fvets.2021.671183

**Published:** 2021-08-10

**Authors:** Catherine Baxevanou, Dimitrios Fidaros, Ilias Giannenas, Eleftherios Bonos, Ioannis Skoufos

**Affiliations:** ^1^Center for Research and Technology – Hellas, Institute for Bio-Economy and Agri-Technology, Volos, Greece; ^2^Laboratory of Nutrition, Faculty of Health Sciences, School of Veterinary Medicine, Aristotle University of Thessaloniki, Thessaloniki, Greece; ^3^Laboratory of Animal Production, Nutrition, and Biotechnology, Department of Agriculture, School of Agriculture, University of Ioannina, Arta, Greece

**Keywords:** energy audit, energy save, renewable energy sources, poultry houses, broiler chicken farms

## Abstract

Broiler facilities consume a lot of energy resulting in natural source depletion and greater greenhouse gas emissions. A way to assess the energy performance of a broiler facility is through an energy audit. In the present paper, an energy protocol for an energy audit is presented covering both phases of data collection and data elaboration. The operational rating phase is analytically and extendedly described while a complete mathematical model is proposed for the asset rating phase. The developed energy audit procedure was applied to poultry chambers located in lowland and mountainous areas of Epirus Greece for chambers of various sizes and technology levels. The energy intensity indices varied from 46 to 89 kWh/m^2^ of chamber area 0.25–0.48 kWh/kg of produced meat or 0.36–1.3 kWh/bird depending on the chamber technology level (insulation, automation, etc.) and the location where the unit was installed. The biggest energy consumer was heating followed by energy consumption for ventilation and cooling. An advanced technology level can improve energy performance by ~ 27%−31%. Proper insulation (4–7 cm) can offer a reduction of thermal energy consumption between 10 and 35%. In adequately insulated chambers, the basic heat losses are due to ventilation. Further energy savings can be achieved with more precise ventilation control. Automation can offer additional electrical energy saving for cooling and ventilation (15–20%). Energy-efficient lights can offer energy saving up to 5%. The use of photovoltaic (PV) technology is suggested mainly in areas where net-metering holds. The use of wind turbines is feasible only when adequate wind potential is available. Solar thermal energy is recommended in combination with a heat pump if the unit's heating and cooling systems use hot/cold water or air. Finally, the local production of biogas with anaerobic fermentation for producing thermal or electrical energy, or cogenerating both, is a choice that should be studied individually for each farm.

## Introduction

The European Union has set the goal of reducing energy consumption and CO_2_ emissions due to high energy prices and the need to achieve sustainable development (1). Broiler houses consume a lot of energy which on the one hand leads to natural source depletion and on the other hand is responsible for greater greenhouse gas emissions (GHGs). Furthermore, GHGs emitted by livestock operations (including broiler houses) along with emitted air pollutants represent potential risks to farmers' health, livestock, and residents in the vicinity. The energy consumption in livestock buildings is expected to increase in the coming years due to increasing levels of mechanization and automation and due to the intensification of livestock production to meet the enlarged nutritional needs of a growing population. On the other hand, the reduction of energy intensity in livestock facilities can help the European Union achieve sustainable development in the near future, introducing green and eco-labeled products into the European market.

The annual energy consumption in livestock buildings concerns (a) the control of internal microclimate (temperature, humidity, air quality, and lighting), (b) the animals' feeding (provision food, medicines, and water), (c) both animal and facility hygiene, and (d) applications related with the production process. In broiler facilities, the basic energy needs are limited to the first two categories. In fact, a broiler house is an enclosed building in which there is complete mechanical control of the microclimate.

Energy crises in the ‘70s induced in the food sector the concepts of primary energy and life cycle analysis (2). The relevant work of the ‘70s and ‘80s is summarized in a review paper (3) in 1989. High energy prices, the upgrade of used equipment, and environmental issues raised by the food production at the beginning of the 21st century resulted in several activities including an evaluation of energy consumption in broiler facilities. In this context, the issue of energy consumption in broiler farms has been addressed in some publications (4–8) which address different locations on earth. According to (9) and (10), in a broiler house, the energy consumption varies between 12 and 16 MJ/t of bird or 60–80 kWh/m^2^.

Energy audits are processes that reveal the most energy-intensive operations and devices of a production unit as well as the energy efficiency of the examined processes and equipment. Thus, energy audits guide veterinarians, engineers, and farmers to choose the most effective energy measures to reduce energy consumption, leading to reduction of natural resource depletion and GHGs. The energy audit concept was initially developed in the US being adopted by Europe, 20 years ago, in many applications. Methodologies have been developed for conducting energy audits in industry and buildings under relevant European Union Directives (from 93/76/EC to 2018/844/EU) (11–15). For conventional buildings, the energy audit methodology is based on the 2002/91/EC (12) directive supported by numerous European and International norms (ENs and ISOs). In the last two decades, the issue of an energy audit in the industry has been addressed by many research and development projects (like FP7, Intelligent Energy and Horizon 2020), however, without any specific directive unless 93/76/EC (12). The issue of energy audit in livestock facilities has not been addressed in Europe at the level of directives as a separate subject. For this reason, it is treated utilizing a combination of methods concerning buildings and industry. In this endeavor, the NRCS/USDA recommended valuable practices, based on energy audits conducted by experts in the USA (16). However, energy efficiency issues of individual processes such as heating and cooling, cogeneration, and energy label and eco-design are covered by relevant EU directives (17–19). In (10), a methodology for energy audits in broiler facilities is presented. However, the process of an energy audit in broiler farms has not been presented in detail yet at a theoretical level with a complete description of the mathematical model used.

Renewable energy source (RES) utilization in broiler farms usually focuses on the utilization of biomass. In this work, in addition to biomass, the use of wind, solar, and geothermal energy will be examined.

The use of wind energy in a poultry house of 22,000 birds in Turkey is examined in (20) according to the yearly electrical energy consumption profile and the available wind potential focusing on electrical energy consumed for lighting and ventilation.

Greater interest has been developed in the use of photovoltaic (PV) to cover electrical loads given the large available area on the roof of broiler farms. (21, 22) examined the use of PV as a stand-alone and interconnected system to meet the needs of a poultry farm with or without storage of electricity in batteries. The same subject is analyzed in (23) using a different approach. In (24), it is proved that the use of PVs in the roof of poultry chambers only slightly aggravates the microclimate inside the chamber. Finally, in (23), a feasibility study for the use of PV in poultry farms is presented.

The use of solar thermal energy for heating in poultry farms requires sophisticated heating systems beyond the usual ones used, such as heated walls, floors, ceilings, and heat exchangers for air heating. Thus, at a research level, passive solar systems with a heated roof (25) or solar walls (26) have been proposed. The most common is the investigation of the utilization of heat that can be abducted from the rear surface of a PV since the incident radiation only by a percentage of 12–18% is converted into electricity while the rest is reemitted as thermal radiation. Thus, the heat utilization by photovoltaic/thermal (PV/T) hybrid systems (26, 27) has been considered. This heat can be used directly or indirectly through a heat exchanger to heat the air of a poultry house. Other thermal solar systems, such as concentrating solar collectors and vacuum solar thermal collectors, are still very abstract and practical progress has been much less (26). Finally, the use of thermal solar energy in collaboration with a heat pump (26) is examined for poultry heating as well as for the enhancement of the operation of anaerobic digestion systems.

Instead, the use of geothermal energy is considered to meet the thermal needs of poultry chambers (28). Apparently, this can be applied only to new units, as in existing facilities it is needed to reconstruct the main buildings. It is important to note that in the case of a geothermal system, the cooling needs during the summer can also be met. Finally, (29) examined the effect of using a geothermal system on bird health.

In this paper, an analysis of broiler houses' energy performance is presented and it is accompanied by energy-saving measures that are suggested according to the findings of this analysis. For the energy analysis, the method of the energy audit is used. For that, a protocol for energy audit in broiler houses is developed and presented for the first time analytically, fully documented, and with a full description of the mathematical model. This protocol is applied in several broiler houses of various technology levels and topographic relief (e.g., mountainous and lowland stations). The findings of these energy audits are presented and analyzed followed by suggested energy-saving measures and renewable energy solutions for the different types of broiler facilities. In addition, some strategies to reduce energy intensity in broiler facilities are suggested according to the type of broiler facility evaluated.

## Methodology

The basic methodology used was the assessment of broiler facilities' energy performance through the procedure of energy audits. An energy audit is a systematic process that aims to (a) form a comprehensive view on the energy consumption profile of a building or system by identifying the factors that affect it, (b) consider energy-saving options taking into account the total cost of the product, and (c) provide a comprehensive proposal with the energy-saving measures that could be implemented. In livestock, the building, its operating strategies, and the electromechanical (EM) systems are examined at the same time.

**Figure 1:**
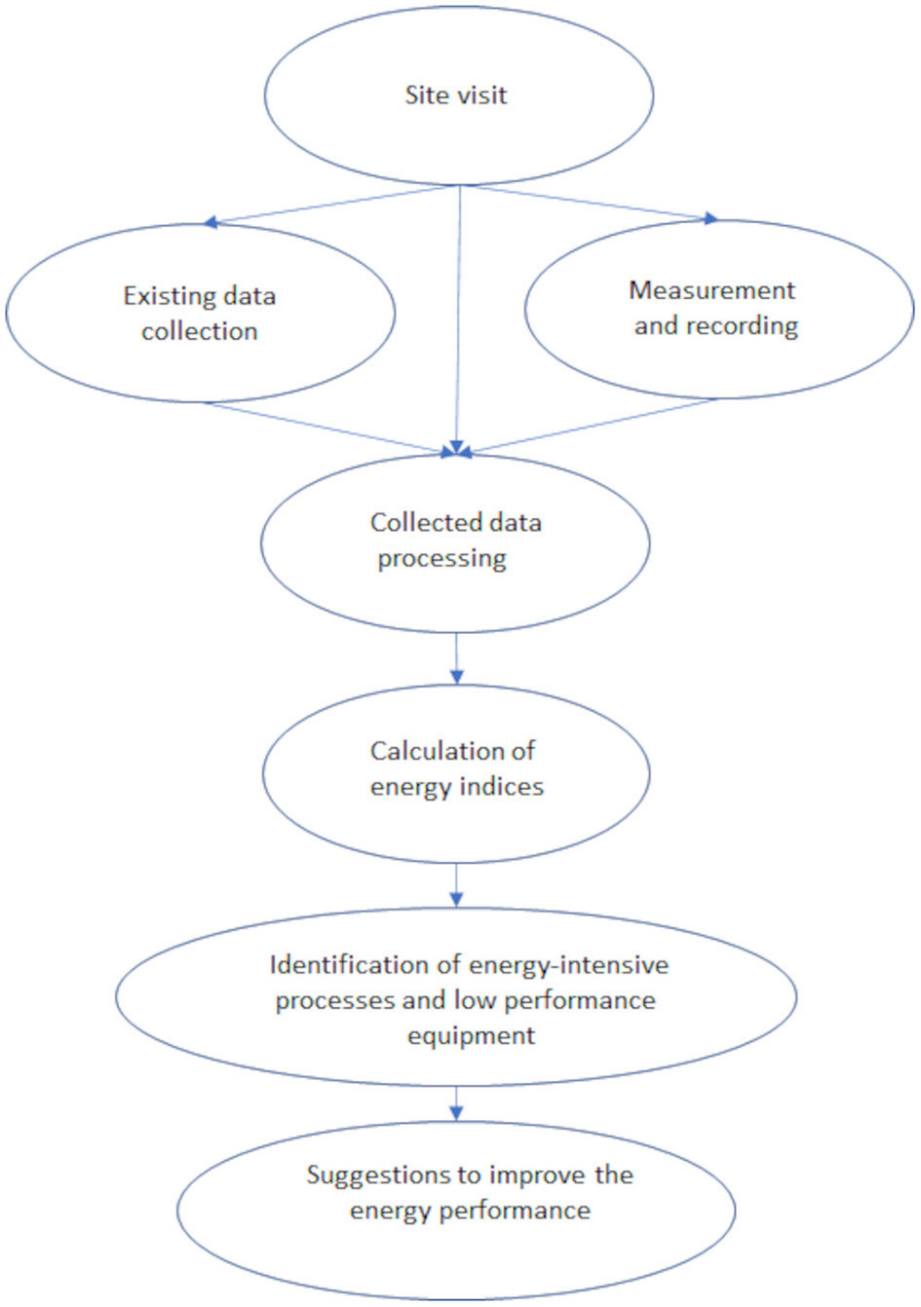
Flowchart of the energy audit procedure.

Energy audit procedures for broiler houses have been suggested and presented by authors in (10, 30). An energy audit consists of two discrete phases. The first concerns an operational rating approach using the data of energy bills and the production data to calculate the energy consumption. From the first phase, the auditor acquires a general perspective about the broiler facility energy performance but analysis is required to be able to (a) distribute the consumed energy among the chambers of broiler facility units or different procedures inside a chamber, (b) allocate the most energy-consuming activities, (c) assess the efficiency of various procedures, and finally (d) suggest energy performance improvement measures. This analysis is realized in the second phase which is an asset rating approach. In Figure 1, a flowchart of the energy audit procedure is presented. It should be noted that the proposed energy audit procedure concerns only the energy consumption and/or production inside the farm.

### Data Acquisition for the Energy Audit

The data acquisition procedure consists of the following: (a) site visit, (b) data collection, and (c) measurements and recording.

#### Site Visit Procedure

In the first step (site visit), the auditor (i) records the installed equipment which consumes energy, (ii) records the construction characteristics, (iii) records the basic characteristics of the surrounding area, and (iv) interviews the unit manager.

The energy demand in a broiler is for (i) food and water supply (terminal motors for the operation of food lines, auger motors for the transfer of food from silos to the chamber, drilling pumps, water pumps), (ii) lighting (lighting fixtures in the chambers, in the vestibule, outdoor), (iii) heating (radiant brooders, local space air heaters, gas boiler), (iv) ventilation (axial exhaust fans, axial recirculation fans, motors for the operation of ventilation slots), (v) cooling (evaporative pads' pumps, evaporative pads' flaps' motor, mist pumps, heat pumps), and (vi) farm management (compressor, power generator, vehicles, air conditions, incinerator, etc.). For the installed energy-consuming equipment, the auditor records the kind of equipment, the nominal power, the number of identical devices, the efficiency performance coefficients, the position where it is sited inside the farm, and finally the operational characteristics.

The construction elements that can be found in a broiler house are walls (exterior or interior), evaporative pads (as part of the buildings' shell), roof, floor, space divider (plastic curtain), and openings (ventilation openings, ventilation windows, security windows, doors, and fans as part of buildings' shell). For each of these elements, except for openings, the following information should be recorded: (i) kind of construction element, (ii) name, (iii) position in the building, (iv) orientation, (v) length, (vi) height or width, (vii) color, and (vii) composition. The composition concerns the different layers of which the construction material is composed. For each layer, the material and the thickness should be recorded. For openings, the following information should be recorded: (i) kind of opening, (ii) name, (iii) construction elements where it belongs, (iv) height from the floor and distance from the beginning of the construction element where it belongs, (v) orientation, (vi) length and height of the opening or diameter, (vii) material, and (viii) color.

Regarding the surrounding area, the auditor should first get the geographical coordinates of the farm position. Then he should record the relative position of the chambers as well as the location of the chambers inside the broiler farm site. For each element that could cause shading (other buildings, parts of the same building, cantilevers, shades, or natural elements, e.g., mountains), the following information should be recorded: (i) type of shading element, (ii) construction element that shades, (iii) dimensions (length, height, or width), and (iv) distance from the construction element that shades. Furthermore, the existence of an element that may alter the local microclimate to what prevails in the general area (e.g., water elements, or elements that block the passage of wind, etc.) and local wind regime should be recorded. Finally, the availability of water and electricity networks should be examined.

Finally, the first step is completed with an interview with the owner or manager of the broiler unit. In this interview, data should be recorded about the (i) owner/manager name and contact info as well as his position in the farm, (ii) data about the poultry farm establishment like the year of construction and renovation, capacity of chambers in birds, and existence of unit's plans, (iii) energy consumption information for the last 3 years, e.g., electricity and gas invoices, (iv) production information for the same period, e.g., number and weight of birds per year, (v) existence of equipment manuals, (vi) operational strategy, (vii) renovation that has taken place, and (viii) interventions that are planned. Specifically about the operational strategy, information should be collected about (i) breeding duration, (ii) time interval between two consecutive breedings, (iii) lighting operation schedule, (iv) heating operating conditions (design temperature each day of the breeding), (v) schedule and operating conditions of feeding and water supply equipment, (vi) fans' operation schedule, (vii) schedule (operating conditions) of cooling equipment, (viii) schedule (operating conditions) of window motors, and (ix) schedule of operation of other machines.

#### Data Collection

In the second step, the auditor should collect data that cannot be recorded by farm inspection. Most of them are collected and delivered by the owner/manager after the interview or during it. These data include (i) construction plans of the chambers and plans of the area, (ii) manuals and technical characteristic specifications of the equipment, (iii) existing energy consumption measurements, (iv) energy consumption invoices, (v) production data in breeding and annual base for the last 3 years (initial number of birds, final number of birds, final weight of birds), and (vi) local climatic data. Energy consumption invoices can have covered the financial data. As far as electricity consumption is concerned, the following info should be gathered on a monthly basis: (i) periods that cover the invoice, (ii) energy consumption, (iii) agreed electrical power, (iv) electrical power demand, and (v) power factor. For the fossil fuel consumption, the auditor records the date of purchase (or the period between two invoices), the purchase quantities in kg (and/or liters), and the specific volume of the fuel. The local climatic data can be collected either by existing measurements from local weather stations or by national databases.

#### Measurement and Recording

The data acquisition phase is completed with measurements. The measurements can be instantaneous or specific in duration. Measurements may give information about the equipment's efficient operation, the materials' properties, and the consumed energy and check whether the equipment used and the applied breeding strategy ensure the required microclimate. Instantaneous measurements for the assessment of equipment efficient operation may concern (i) the burners' efficiency with exhaust gas analysis, (ii) the exhaust fans' operation concerning the airflow rate and the pressure drop with differential manometer and/or pitot tube and/or hotwire anemometer, (iii) evaporative pad operation with differential manometer, and (iv) heat losses from tubes with infrared laser thermometers. Long time measurements may concern (i) the thermal transmittance of a construction element with a combination of heat flux meters and differential thermometers, (ii) the electrical energy consumption of the farm or a specific device and electrical power quality with an electricity analyzer, (iii) the fuel consumption with a flow meter, etc. Measurements concerning the quality of achieved microclimate may include (i) lighting level at the bird height with a lux meter, (ii) air quality (temperature, relative humidity, and CO_2_ concentration) with an air quality meter, (iii) airspeed at the birds' level and at the fans' level with a hotwire anemometer, (iv) surfaces' temperatures with an infrared camera or with infrared laser thermometer or with contact thermometers, and (v) noise levels. Finally, external area climatic conditions during the measurements should be recorded.

### Data Analysis

The data analysis phase is constituted by four steps: (a) processing of the collected data, (b) calculation of energy indices, (c) identifying energy-intensive processes and low-performing equipment, and (d) suggestions for energy performance improvement.

#### Collected Data Processing

The processing of the collected data includes (i) collection of installed power data, (ii) distribution of installed power by type of consumption and chambers, (iii) elaboration of time series of energy consumption and production, (iii) calculation of operating hours of the individual devices, and (iv) distribution of energy consumption per type of consumption and chamber.

The installed power data are organized in tables according to the chamber of the farm where they belong and according to the type of consumption. The installed power is distinguished among thermal and electrical power. The thermal power is distinguished among thermal power for heating and thermal power for motion (vehicles' operation). The electrical power is distinguished to (i) feeding and water supply, (ii) lighting, (iii) heating, (iv) ventilation, (v) cooling, and (vi) other equipment.

**Figure 2:**
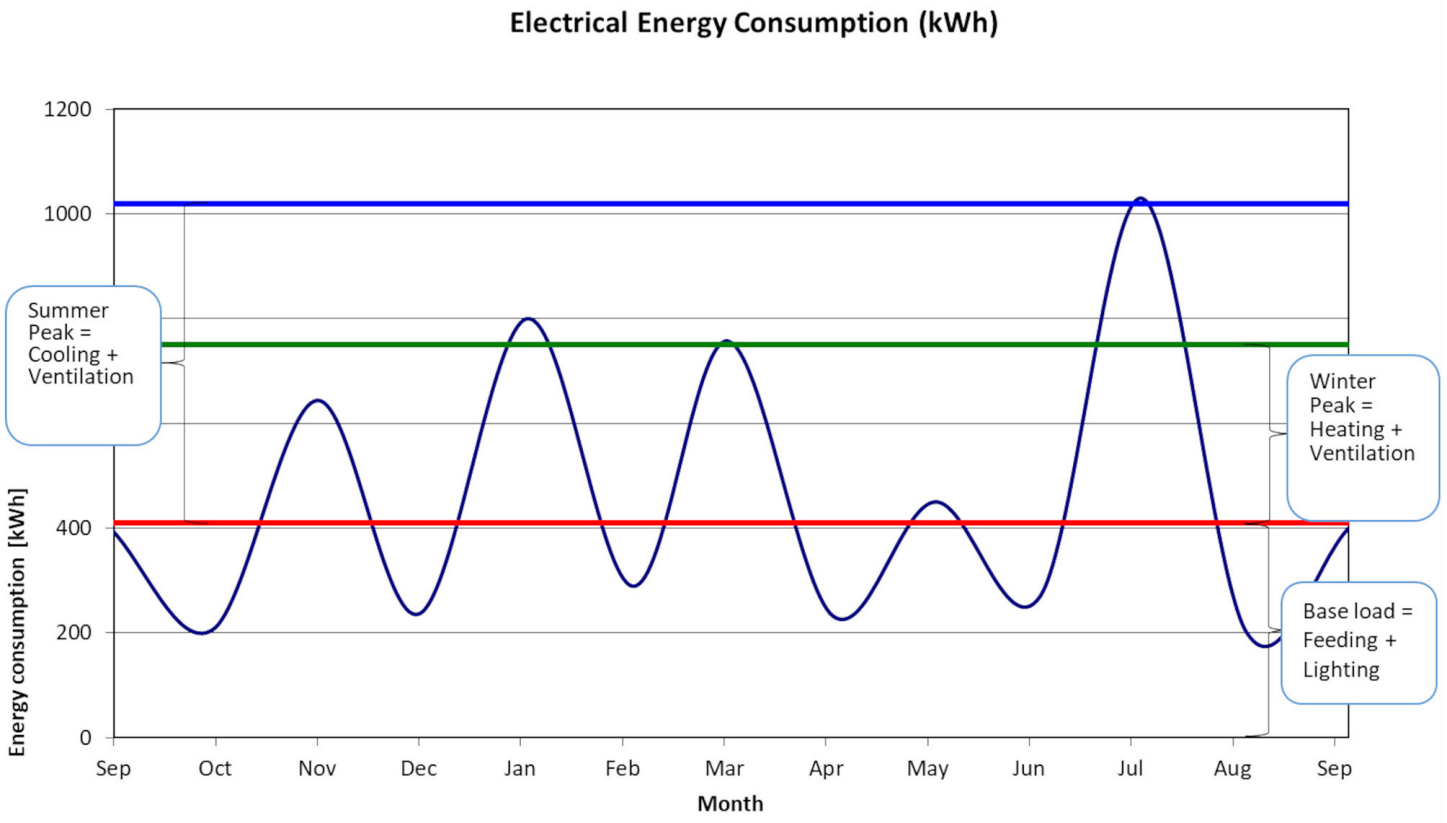
Yearly pattern of electrical energy consumption in broiler farms.

The production data are used for the creation of time series of production. The energy consumption data are also used for the creation of time series on a monthly basis. After elaboration of 3 years of data, the basic yearly pattern is determined, as shown in Figure 2 where the monthly energy consumption is presented. This pattern along with analytical calculations about the theoretical energy consumption is used for the determination of operation hours of each device.

For the calculation of operating hours, an asset-rating approach with several assumptions is used. The basic assumption is that the equipment operates in its nominal capacity and succeeds to achieve the desired internal microclimate conditions. In the pattern shown in Figure 2, a base load and two peaks (winter and summer) are recognized.

(1) $$\begin{array}{r} {\text{E}}_{\text{b}\text{l},\text{m}}\;=\;{\text{E}}_{\text{f}+\text{w},\text{m}}+{\text{E}}_{\text{l},\text{m}} \end{array}$$

where E_bl, m_ (kWh/m) is the average monthly lower energy consumption. Two difficulties exist in the calculation of the base load. The first is that the breeding is not continuous and the second is that in a broiler farm the breedings among the different chambers are not synchronized. E_l, m_ (kWh/m) is the average monthly energy consumption for lighting, and E_f+w, m_ is the average monthly energy consumption for feeding and water supply.

The total yearly energy consumption for lighting, E_l_ (kWh), can be calculated directly from the installed power and the standard daily lighting schedule. According to the schedule, the energy consumption is calculated from Equation (2).

(2) $$\begin{array}{r} {\text{E}}_{\text{l}}\;=\;{\text{n}}_{\text{y}\text{b}}\left(\underset{\text{i}}{\sum }{\text{t}}_{\text{i}}{\text{P}}_{\text{l}}+\underset{\text{i},\text{a}\text{u}\text{x}}{\sum }{\text{t}}_{\text{i},\text{a}\text{u}\text{x}}{\text{P}}_{\text{l},\text{a}\text{u}\text{x}}\right) \end{array}$$

where *n*_yb_ (27) is the number of breedings during the year, *i* is the number of breeding days, t_i_ [h] is the time of lighting operation during the *i* day, P_l_ is the installed lighting power inside the broiler chamber, t_i, aux_ is the daily time of operation of auxiliary lighting (lobby and exterior lighting), and P_l, aux_ is the installed power of auxiliary lighting. The average monthly energy consumption for lighting is calculated from the following equation.

(3) $$\begin{array}{r} {\text{E}}_{\text{l},\text{m}}\;=\;\frac{{\text{E}}_{\text{l}}}{\left(\frac{\left({\text{n}}_{\text{y}\text{b}}{\text{n}}_{\text{b}}\right)}{30}\right)} \end{array}$$

where *n*_*b*_ [days] is the duration of each breeding in days. The monthly average energy for feeding and water is calculated from the following equation.

(4) $$\begin{array}{r} {\text{E}}_{\text{f}+\text{w},\text{m}}\;=\;{\text{E}}_{\text{f},\text{m}}+{\text{E}}_{\text{w},\text{m}} \end{array}$$

where E_f, m_ is the monthly energy consumption for the operation of feeding equipment and E_w, m_ is the monthly average energy consumption for water supply. It is assumed that the feeding equipment operates automatically securing food and water at demand. It is assumed that it operates for 6 h per day (31). Thus, the monthly energy consumption for feeding is calculated as

(5) $$\begin{array}{r} {\text{E}}_{\text{f},\text{d}}\;=\;{\text{t}}_{\text{f}}{\text{P}}_{\text{f}} \end{array}$$

where t_f_ [h] is the monthly hours of operation of feeding equipment and P_f_ (25) is the installed power of feeding equipment. From the combination of Equations 1–5, the average daily energy consumption for water, E_wd_ (kWh/d), supply can be derived. Finally, the daily and yearly hours of operation of water supply equipment can be calculated as follows:

(6) $$\begin{array}{r} t_{w,d}\;=\;\frac{E_{w,d}}{P_{w}} \end{array}$$

where t_w, d_ (h) is the monthly average hours of operation of water supply equipment and Pw (25) is the installed power of water supply equipment.

The difference between the base load and the winter peak corresponds to the energy consumed for heating and ventilation.

(7) $$\begin{array}{r} {\text{E}}_{\text{w},\text{p},\text{m}}-{\text{E}}_{\text{b}\text{l},\text{m}}\;=\;{\text{E}}_{\text{v},\text{m}}+{\text{E}}_{\text{h},\text{m}} \end{array}$$

where E_w, p, m_ (kWh/m) is the monthly average energy consumption peak during the winter, E_v, m_ (kWh/m) is the monthly energy consumption for ventilation, and E_h, m_ is the monthly average energy consumption for heating.

The yearly thermal energy consumption E_h, th, y_ (kWh) for heating can be calculated analytically with an hourly step according to ISO 13790 (32) with the following assumptions: (a) the effect of dynamic phenomena related to heat storage is ignored since the ratio area/volume is small and the heat capacity of the construction materials is low, (b) thermal gains are considered fully exploitable by setting their utilization heat gain coefficient unity, (c) direct solar gains are not taken into account since during the operation of the broiler house the openings are closed while the existence of insulation prevents the indirect solar thermal gains, and (d) thermal gains due to equipment operation are not taken into account. This requires the calculation of (i) thermophysical properties of construction elements (33), (ii) the average heat transfer coefficient, U_m_, (iii) the hourly variation of external temperature during a typical day of breeding (one for each of the five breedings per year), (iv) chickens' thermophysical properties and emission for each day of the breeding (34, 35), and (v) ventilation needs (10, 30). Finally, the yearly hours of operation of the heating equipment, t_h, y_ (h), is calculated.

(8) $$\begin{array}{r} {\text{t}}_{\text{h},\text{y}}\;=\;\frac{{\text{E}}_{\text{h},\text{t}\text{h},\text{y}}}{{\text{P}}_{\text{h},\text{t}\text{h}}} \end{array}$$

where P_h, th_ (25) is the thermal installed power for heating. The corresponding yearly electrical energy consumption can be calculated from the following relationship:

(9) $$\begin{array}{r} {\text{E}}_{\text{h},\text{y}}\;=\;{\text{t}}_{\text{h},\text{y}}{\text{P}}_{\text{h},\text{e}} \end{array}$$

For the calculation of the monthly average energy consumption for heating, the heating period, t_h, p_ [months], should be calculated according to ISO 13790:

(10) $$\begin{array}{r} {\text{E}}_{\text{h},\text{m}}\;=\;\frac{{\text{E}}_{\text{h},\text{y}}}{{\text{t}}_{\text{h},\text{p}}} \end{array}$$

Then the monthly average energy consumption for ventilation can be calculated from Equation (7). Then, the monthly operation hours of ventilation equipment, t_v, m_ (h), can be calculated by

(11) $$\begin{array}{r} {\text{t}}_{\text{v},\text{m}}\;=\;\frac{{\text{E}}_{\text{v},\text{m}}}{{\text{P}}_{\text{v},\text{m}}} \end{array}$$

This can be compared with the info taken from the interview about the ventilation operation strategy. If important discrepancies are observed, then it should be calculated whether the installed equipment is adequate for the supply of necessary fresh air. According to the conclusions of the results, the auditor will calibrate the operational hours either of ventilation or of heating.

The difference between the base load and the summer peak corresponds to the energy consumed for cooling and ventilation.

(12) $$\begin{array}{r} {\text{E}}_{\text{s},\text{p},\text{m}}-{\text{E}}_{\text{b}\text{l},\text{m}}\;=\;{\text{E}}_{\text{v},\text{m}}+{\text{E}}_{\text{c},\text{m}} \end{array}$$

where E_s, p, m_ (kWh/m) is the monthly average energy consumption peak during the summer, E_v, m_ (kWh/m) is the monthly energy consumption for ventilation, and E_c, m_ is the monthly average energy consumption for cooling. From Equation (12), the monthly average energy consumption for cooling can be calculated. Then, the cooling period t_c, p_ (months) will be calculated according to ISO 13790. Finally, the yearly energy consumption for cooling will be calculated according to

(13) $$\begin{array}{r} {\text{E}}_{\text{c},\text{y}}\;=\;{\text{t}}_{\text{c},\text{p}}{\text{E}}_{\text{c},\text{m}} \end{array}$$

This can be compared with the theoretical energy consumption for cooling. Differences may be due to the inability to meet the requirements of the indoor microclimate. Care should be taken when final energy consumption is calculated through energy demand, and the relevant efficiency coefficients should be taken into account.

When the distribution of electricity consumption is done between cooling, feeding and water supply, heating, lighting, and ventilation, there is always a difficulty in classifying the operation of the fans. We know that fans supply fresh air but at the same time for important periods they are also used for cooling. Based on the cooling base temperature and the climatic data of the areas, it can be considered that the fans operate by 35% for cooling and by 65% for ventilation. Alternatively, the auditor may distribute electricity consumption according to the appliances being consumed and not according to the use being served.

#### Energy Audit Results' Presentation

Since the energy consumption in the level of individual chambers and application has been calculated, the results are presented in terms of (i) energy distribution pies and (ii) energy indices.

The total energy consumption may be distributed among the farms' different chambers. Chambers may also be grouped according to their technology level, their average heat transfer coefficient, *U*_m_, and age. Then thermal and electrical energy consumption may be distributed separately among chambers and/or among groups of them and/or among different uses. These distributions usually are presented in the form of pies.

An additional expression of the results is the calculation of energy indices (e.g., energy consumption per selected unit). Energy indices may concern the total energy consumption in the whole farm and/or on grouped chambers, separately the thermal and electrical energy in the whole farm and/or in grouped chambers and/or in specific uses, and finally primary energy consumption. The unit for which the energy indices are calculated may be the chambers area square meter, the number of the birds, and the weight of the birds.

#### Identification of Energy-Inefficient Processes and Equipment

From the above analysis, the high energy-consuming processes are revealed. These processes will attract our interest in the planning of proposed interventions. Furthermore, information about the individual equipment operation may be drawn from the measurements. Finally, the calculated energy indices may be assessed by comparison to each other or according to international literature values. This will reveal the inefficient processes and inefficient equipment.

#### Suggestions to Improve Energy Performance

The energy audit is completed with the preparation of proposals for the improvement of the energy performance of the broiler unit. Improvement proposals should be categorized into three levels: (i) low cost, (ii) medium cost, and (iii) high cost. They should be accompanied by calculations—assessment of energy improvement—so that their effectiveness can be costed.

For poultries, optimization suggestions may have three general directions:

- In the case of high thermal energy consumption, chamber insulation is recommended mainly if the roof is not insulated or it is poorly insulated.
- In the case of well-insulated chambers, the following interventions should be considered: (a) correct dimensioning of electromechanical Equipment, (b) system efficiency coefficients, and (c) application of automation systems.
- The operation strategy should be considered in collaboration with a specialized zoo technician in terms of breeding seasons and internal microclimate design conditions.

## Study Case

The above-described energy audit protocol was applied for the energy performance assessment of eight broiler farms (with 25 chambers) of various sizes, ages, and technology levels located in lowland and mountainous areas in West Greece. The examined farms belong to two of the biggest broiler cooperatives in Greece. An attempt was made to select units that cover all types of units based on size and technology used in lowland and mountainous areas. Specifically, the following were examined: (a) two (2) large (of equal capacity) farms, one lowland and one mountainous, with seven (7) chambers each (three chambers of new technology and four chambers of old technology for the lowland, four chambers of new and three chambers of old technology for the mountain), (b) two small farms, one lowland with one chamber and one mountainous with three chambers, (c) one mountainous farm with only one chamber old technology, (d) two farms with chambers of only new technology, one mountainous and one lowland with one chamber each, and (e) one mountainous farm with three chambers of mixed technology. Table 1 describes the basic characteristics of examined farms.

**Table 1:** Description of the audited broiler facilities.

**Farm type**	**Location**	**Capacity (number of birds)**	**Chamber's area (m^**2**^)**	**Number of chambers (-)**	**Yearly production (birds/year)**	**Yearly production (kg/year)**
Big farm	Lowland^*^	115,170	7,723	7	532,733	1,472,816
Small farm	Lowland	61,000	3,740	3	324,100	776,544
Only new technology^**^	Lowland	25,000	1,404	1	119,218	273,009
Big farm	Mountainous^*^	88,000	6,345	7	347,666	828,140
Mixing old and new technology	Mountainous	24,000	1,641	3	128,253	326,071
Only old technology^***^	Mountainous	30,000	1,933	2	157,331	384,486
Small farm	Mountainous	20,000	1,253	1	102,994	251,277
Only new technology	Mountainous	20,000	1,264	1	101,509	249,619

* *Lowland and mountainous locations are explained in Table 2 and in the study case description*.

** *New technology refers to well-insulated chambers with automatic control of internal microclimate*.

*** *Old technology refers to purely insulated chambers without automatic control of internal microclimate*.

The lowland area is at sea level with an average latitude of 39°, where the heating degree days are 1,313 (HDD with a base temperature 18.3°C) and the cooling degree hours are 3,399 (CDH with base temperature of 26°C). The mountainous area is considered with an average elevation of more than 500 m at almost the same latitude, with HDD = 2,037 and CDH = 1,694 (36). This means that mountainous areas have almost twice the need for heating and half the need for cooling compared to the lowland areas. Available total solar radiation at the horizontal plane varies from 56.2 to 219.1 kWh/m^2^ for the lowland areas with a clearness index of 0.54 and from 45.1 to 212 kWh/m^2^ for mountainous areas with a clearness index of 0.49 (36). Climatic data are summarized in Table 2. In all the examined units, food and water are supplied automatically “at demand.” Units characterized as “new technology” have chambers with sufficient insulation (with average heat transfer coefficient, U_m_, smaller than 0.71 W/m^2^K for the lowland chambers and 0.58 W/m^2^K for the mountainous chambers) and operation for heating and cooling automated according to the desired internal climate conditions. Old technology is characterized as units with no or insufficient insulation (with average values of U_m_ in the order of 1 W/m^2^K) and operation of heating and cooling without taking into account the internal climatic conditions.

**Table 2:** Climatic data summary for lowland and mountainous farms.

**Climatic parameter**	**Lowland farms**	**Mountainous farms**
Elevation (m)	0 (sea level)	>500
Heating degree days with base temperature 18°C	1,313	2,037
Cooling degree hours with base temperature 26°C	3,399	1,694
Total solar radiation at horizontal plane per month [kWh/m^2^mo]	56–219.1	45.1–212
Clearness index (-)	0.54	0.49

In Table 3, the installed power is given in terms of total power per square area of chambers and per bird capacity for lowland and mountainous farms and separately for thermal and electrical power.

**Table 3:** Installed power in the broiler facilities examined.

**Power**	**Installed power/area (kW/m** ^****2****^ **)**			**Installed power/birds capacity (kW/bird)**		
	**Min**	**Max**	**Average**	**Min**	**Max**	**Average**
Lowland^*^						
Total power	0.18	0.38	0.27	0.011	0.022	0.016
Thermal power	0.15	0.37	0.24	0.009	0.021	0.015
Electrical power	0.01	0.03	0.03	0.001	0.002	0.001
Mountainous^*^						
Total power	0.17	0.32	0.25	0.011	0.020	0.017
Thermal power	0.16	0.30	0.24	0.01	0.019	0.016
Electrical power	0.01	0.03	0.01	0.001	0.002	0.001

* *Lowland and mountainous locations are explained in Table 2 and in the study case description*.

**Figure 3:**
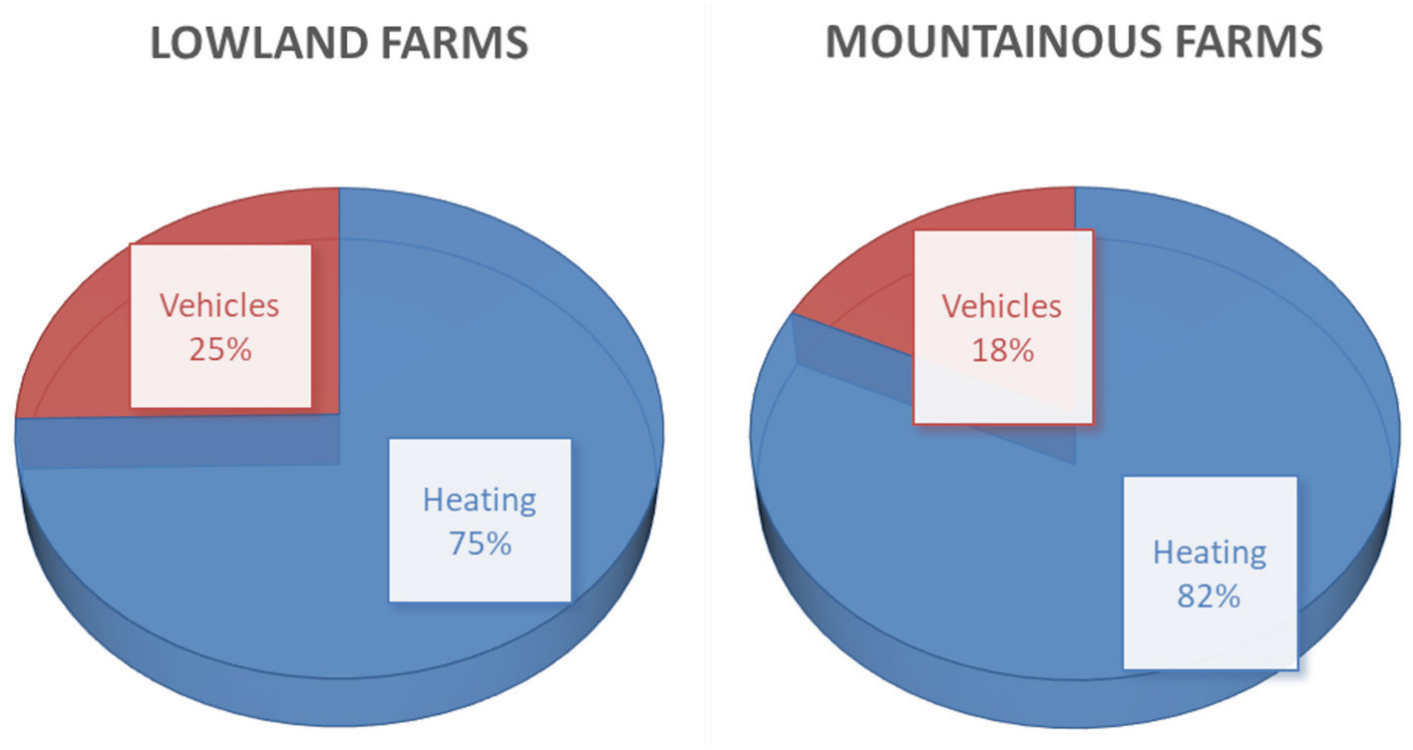
Installed thermal power distribution for lowland and mountainous farms.

From the above, it is clear that the major contribution in installed power comes from thermal power since it represents 91 and 94% for the lowland and mountainous farms, respectively. The thermal power is analyzed to heating and vehicles, as shown in Figure 3. In both lowland and mountainous farms, heating represents the highest ration of installed thermal power.

**Figure 4:**
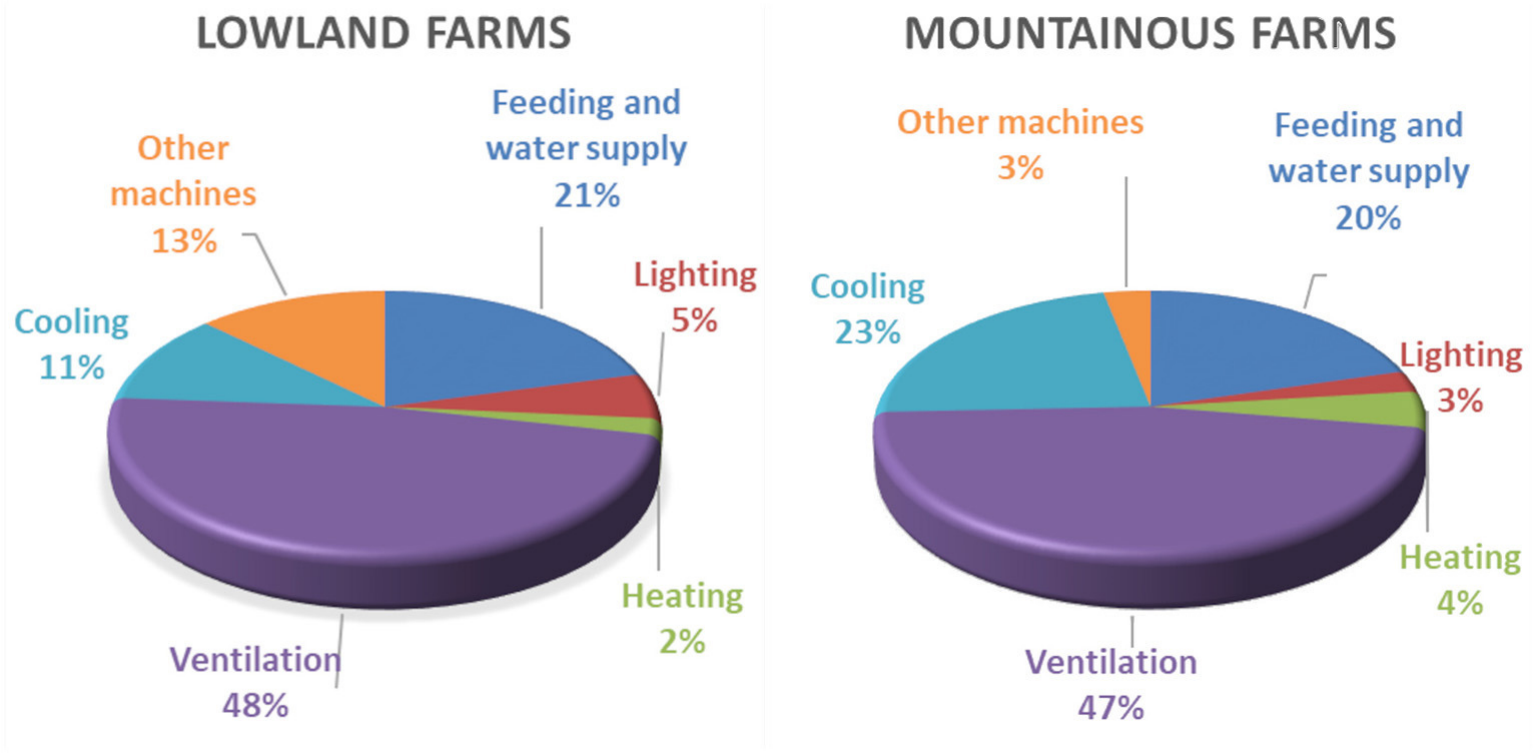
Installed electrical power distribution in lowland and mountainous farms.

The distribution of installed electrical power is presented in Figure 4. In both lowland and mountainous farms, fans represent almost half of the installed electrical power. It should be noted that fans are used not only for ventilation but also for cooling. The rest of the cooling equipment represents 11% in lowland and 23% in mountainous electrical installed power. Feeding requirements cover 20%, and the rest of the installed power concerns lighting and other machines (e.g., compressor). Nevertheless, the installed electrical power is bigger in lowland farms than in mountainous farms due to the increased needs for cooling.

Indicative time series of propane and electrical energy consumption for the lowland and mountainous big farms have been presented in (10).

### Installed Equipment for Food and Water Supply

In all the examined units, the feeding of the birds is done automatically, depending on the level of food in the feeders, through screws that lead the food to the feeders following a path along with the chamber. Depending on the width of the chamber, there are three or four screws driven by motors mounted on one end of the chamber—terminal motors with a power of 0.23–1.12 kW. For the transfer of food from the storage silos (outside the chambers) into the chambers, other screws are used that also work with motors—silo motors, usually one in each chamber with a power of 0.55–2 kW.

### Installed Equipment for Lighting

The energy consumption for lighting mainly concerns the necessary level of lighting inside the chambers to ensure the growth of the birds. For this reason, 11–24-W energy-saving lamps, with >60 lm/W efficiency, or 11–72-W fluorescent lamps are mainly used. Secondarily, lighting is used in the antechambers, when they exist, for auxiliary work. There, a variety of luminaires are used from energy-saving lamps 11–14 W, incandescent lamps 60–160 W, and halogen lamps 125 W. The chamber lighting operates either manually or with a timer, while the auxiliary lighting operates always manually on demand.

### Installed Equipment for Heating

Three types of heating devices were found: (a) fan heaters with thermal power from 50 to 120 kW, (b) brooders with thermal power from 10 to 14 kW, and (c) at one case a gas air boiler of 217 kW. In all cases, the main energy source is propane while in the case of fan heaters and gas boiler there are small electrical consumptions of 0.15–1 kW.

### Installed Equipment for Ventilation

All the fans found to be used for ventilation were axial and can be divided into three categories: (a) exhaust fans mounted on the small side of the chamber, opposite of the evaporative pads (when they exist), (b) exhaust fans mounted in the large side of the chamber, and (c) recirculation fans inside the chamber. Exhaust fans are of 0.55–1.12 kW with diameter varying from 0.5 to 1.25 m and flow rate from 3,000 to 36,000 m^3^/h. Recirculating fans are 0.1–0.37 kW. In addition to the fans, in high-technology units, for the operation of ventilation, there are also small motors that open and close the ventilation openings with electrical power from 0.12 to 0.8 kW.

### Installed Equipment for Cooling

The basic technology used for air conditioning, in the examined broiler farms, is that of evaporative cooling and is carried out either with evaporative pads or with evaporator air coolers. For the operation of evaporative pads, pumps of electrical power from 0.4 to 1.5 kW are used to circulate water, which is the basic energy consumption. In addition, lower consumptions concern the movement of the evaporative pads' flaps made with motors of 0.12–0.55 kW. Evaporator coolers as compact devices were of 2.2–2.5 kW electrical power.

## Results

After the elaboration of energy audit data according to the described methodology, the energy consumption is calculated at the chamber and process levels.

### Energy Consumption Distribution

**Figure 5:**
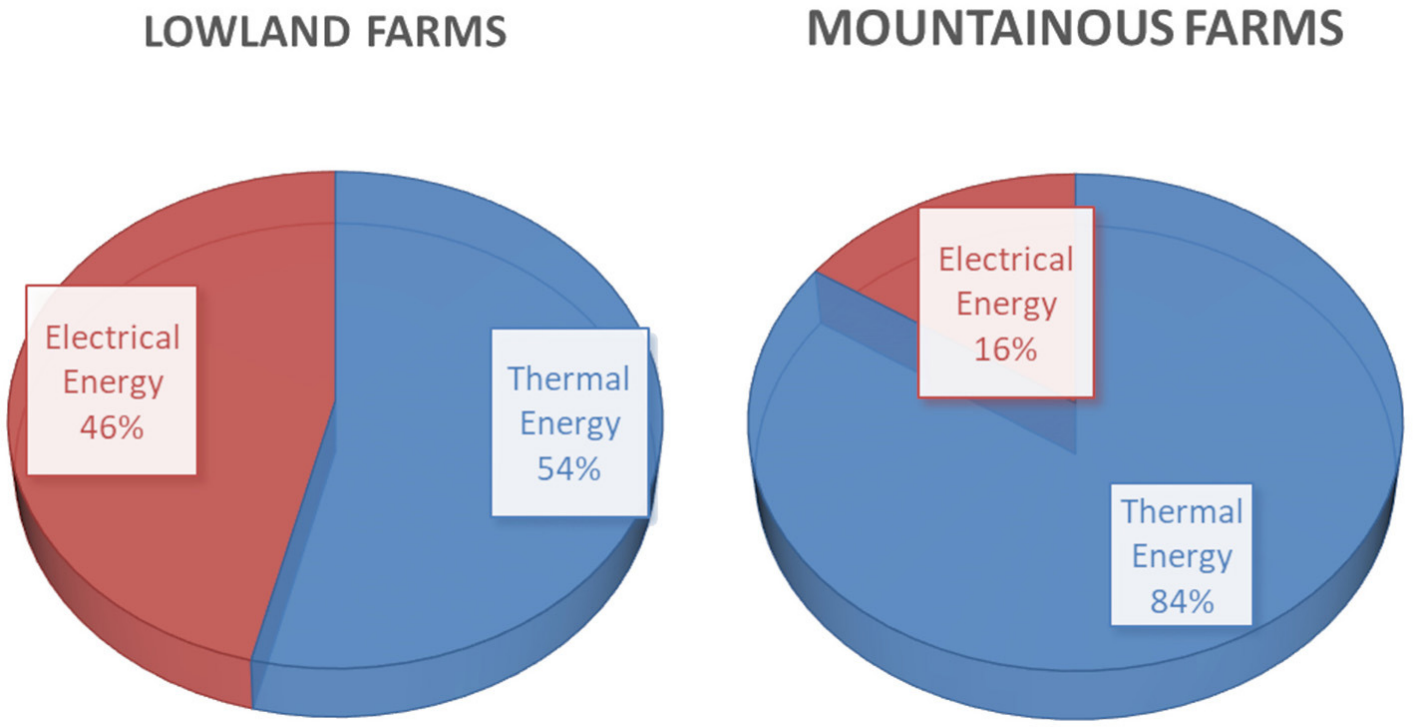
Distribution of total final energy consumption in lowland and mountainous farms.

In Figure 5, the distribution among thermal and electrical energy is given for lowland and mountainous farms. Although in both lowland and mountainous farms the percentage of electrical power was small, the final energy consumption pattern reveals two different responses. In lowland farms, the electrical energy is 46% of the final energy consumption while in the mountainous farms the percentage of electrical energy is only 16%. It is obvious that in mountainous farms the high energy demand is related to heating needs while in lowland farms cooling needs are equally important.

**Figure 6:**
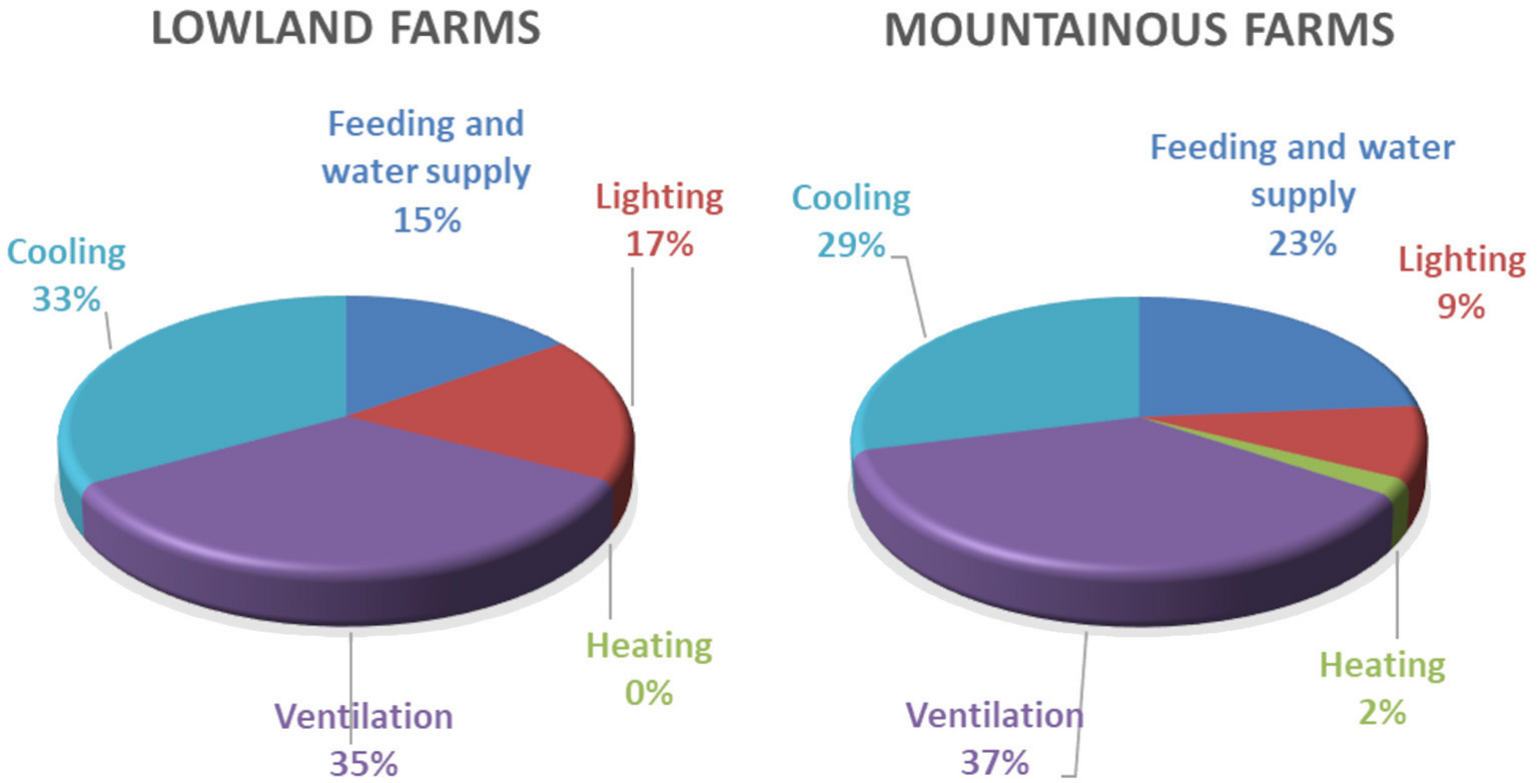
Distribution of electrical energy consumption in lowland and mountainous farms.

In Figure 6, the distribution of electrical energy among the served processes is given for lowland and mountainous farms. The pattern of distribution is similar with small differences in the percentages of cooling and feeding energy consumption. For cooling, about 30% of the electrical energy is consumed. Ventilation represents the biggest consumer since it operates during the whole year. Feeding is the third consumer followed by lighting.

**Figure 7:**
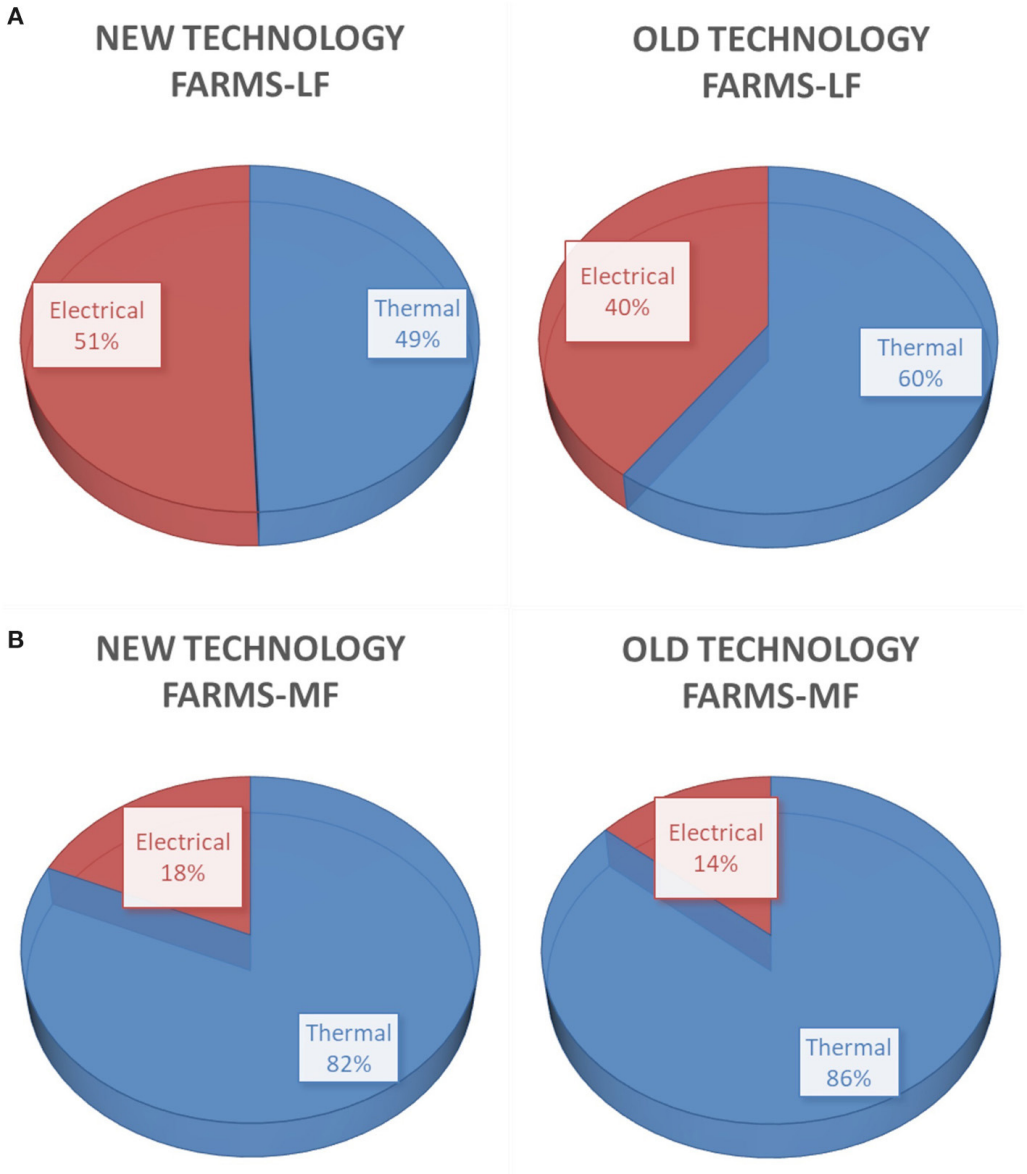
Distribution of total final energy consumption **(A)** in lowland farms and **(B)** in mountainous farms, for new and old technology chambers.

In Figure 7A, the distribution among thermal and electrical energy in lowland farms is given for new and old technology chambers. In the lowland farms, where the cooling loads are important, inefficient cooling technologies lead to increased electrical energy consumption.

In Figure 7B, the distribution of thermal and electrical energy in mountainous farms is given for new and old technology chambers. In mountainous farms, the big consumer is heating. Small differences are observed in the distribution among old and new technology chambers attributed to poorer electromechanical equipment.

**Figure 8:**
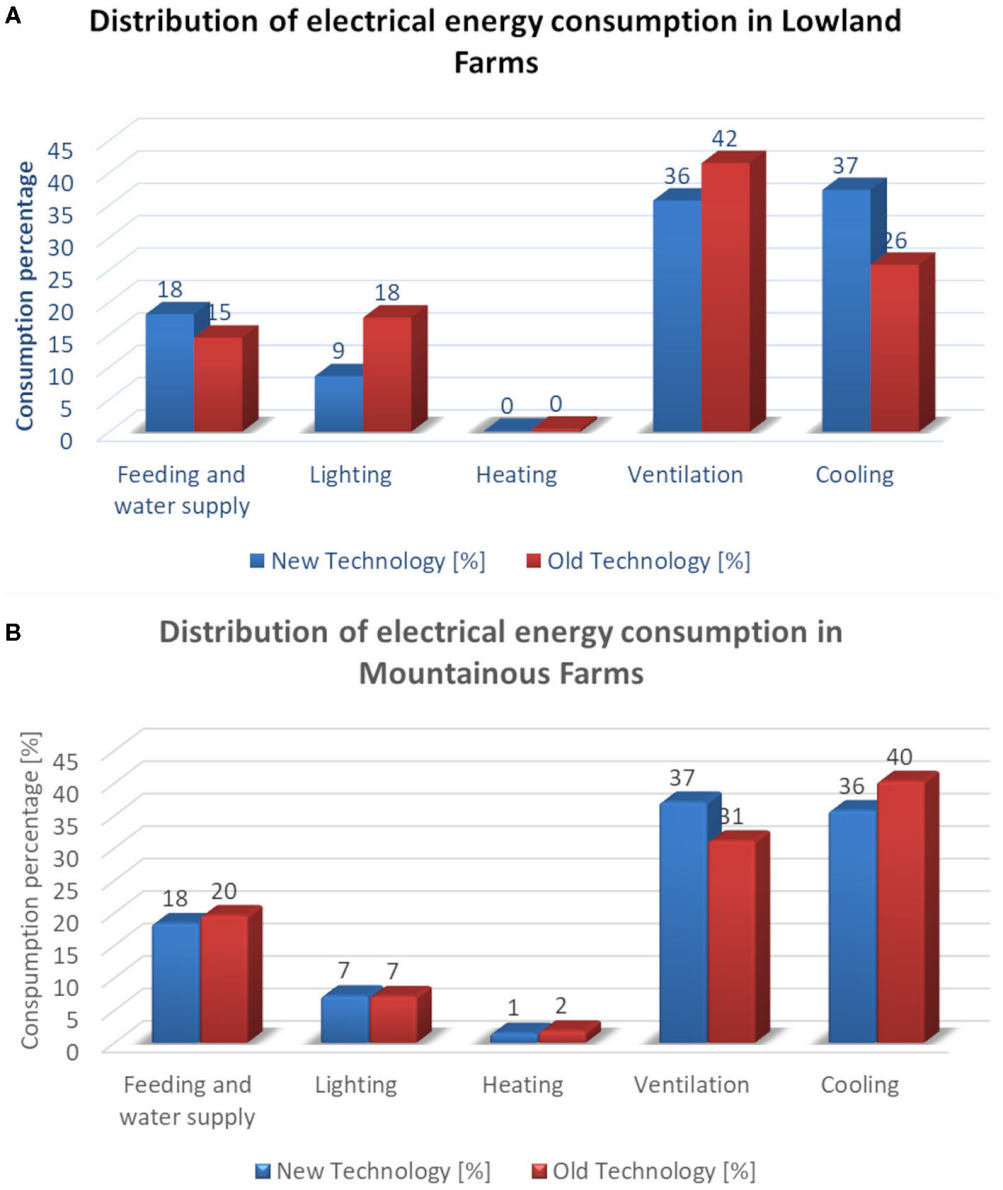
Distribution of electrical energy consumption **(A)** in lowland farms and **(B)** in mountainous farms, for new and old technology chambers.

In Figure 8A, the distribution of electrical energy among the served processes in lowland farms is given for new and old technology chambers. In new technology chambers, cooling is comparable with ventilation. In old technology level, ventilation share is much more important than cooling share since ventilation is widely used for temperature control. In new technology chambers, the use of energy-efficient lights leads to important energy saving.

In Figure 8B, the distribution of electrical energy among the served processes in mountainous farms is given for new and old technology chambers. The electrical energy distribution profile for new technology chambers in mountainous farms is almost the same as the distribution in lowland farms except the appearance of a small share of electrical energy consumption for heating. As far as the old technology chambers are concerned, the increased share of cooling is attributed to low efficient equipment.

### Energy Indices

In Table 4, the energy indices concerning final energy consumption are presented for lowland and mountainous farms, for old and new technology chambers. The presented energy indices are (i) final total energy consumption per chamber area, (ii) final total energy consumption per bird, and (iii) final total energy consumption per produced meat weight. For each index, three values are given: the average, the minimum, and the maximum. The lower energy consumption is achieved to lowland farms using new technology while the worst performance is met in the mountainous farms with old technology. This is expected since the higher energy consumer is the heating and mountainous farms with insufficient insulation have a big energy demand. Nevertheless, lowland farms with old technology have comparable energy indices with mountainous farms with new technology. This means that there is energy-saving potential in electrical consumption as well.

**Table 4:** Energy indices according to the final energy consumption of the examined broiler facilities.

**Energy index**	Old technology^***^– lowland^*^			Old technology– mountainous^*^			New technology^***^– lowland			**New technology– mountainous**		
	**Max**	**Min**	**Av**	**Max**	**Min**	**Av**	**Max**	**Min**	**Av**	**Max**	**Min**	**Av**
Final energy consumption/area (kWh/m^2^)	89.4	52.48	67.54	131.56	74.63	96.59	61	30.15	46.38	106.26	54.64	70.72
Final energy consumption/bird (kWh/bird)	1.30	0.79	0.99	1.27	0.68	1.05	0.89	0.36	0.73	1.29	0.83	0.99
Final energy consumption/weight (kWh/kg)	0.47	0.29	0.37	0.52	0.27	0.43	0.32	0.15	0.25	0.53	0.35	0.41

* *Lowland and mountainous locations are explained in Table 2 and in the study case description*.

** *New technology refers to well-insulated chambers with automatic control of internal microclimate*.

*** *Old technology refers to purely insulated chambers without automatic control of internal microclimate*.

Before proceeding to the discussion of energy audit findings, another issue should be considered. This is related to the quality of consumed energy. Electrical energy is expensive energy in terms of “primary energy” consumption. In (10), authors had presented energy indices, concerning energy consumption per produced meat weight and per broiler house area, split into thermal and electrical energy and finally energy indices according to primary energy consumption. The energy indices concerning the consumed energy per bird for lowland and mountainous units with old and new technology are presented in Table 5. Nevertheless, when this index is given, it should be accompanied by information about birds' final weight. In the examined cases, final weight varies between 2.4 and 2.8 kg per bird, depending on the time period and the broiler house location.

**Table 5:** Energy indices according to the primary energy consumption of the examined broiler facilities.

**Chamber location/technology level**	**Energy index**	**Final energy per bird (kWh/bird)**	**Primary energy per bird (kWh/bird)**
Lowland^*^-new technology^**^	Thermal energy	0.36	
	Electrical energy	0.37	
	Total energy	0.73	1.45
Lowland–old technology^***^	Thermal energy	0.60	
	Electrical energy	0.39	
	Total energy	0.99	1.76
Mountainous^*^-new technology	Thermal energy	0.80	
	Electrical energy	0.19	
	Total energy	0.99	1.39
Mountainous–old technology	Thermal energy	0.90	
	Electrical energy	0.15	
	Total energy	1.05	1.38

* *Lowland and mountainous locations are explained in Table 2 and in the study case description*.

** *New technology refers to well-insulated chambers with automatic control of internal microclimate*.

*** *Old technology refers to purely insulated chambers without automatic control of internal microclimate*.

Finally, in Table 6, energy indices are presented in terms of final use for the examined cases for final energy consumption and primary energy consumption. There are four uses of energy consumption: (a) feeding, (b) lighting, (c) heating, and (d) cooling and ventilation. It should be noted that energy for heating is different than thermal energy presented earlier since it contains both thermal and electrical energy consumed for heating.

**Table 6:** Energy indices according to the energy usage of the examined broiler facilities.

**Chamber location/technology level**	**Energy index**	**Final energy per kg (kWh/kg)**	**Final energy per area (kWh/m^**2**^)**	**Primary energy per kg (kWh/kg)**	**Primary energy per area (m^**2**^)**
Lowland–new technology	Feeding	0.02	3.48	0.06	10.09
	Lighting	0.01	1.65	0.03	4.88
	Heating	0.14	26.25	0.18	32.84
	Cooling and ventilation	0.08	15	0.23	43.5
Lowland–old technology	Feeding	0.02	3.61	0.06	10.47
	Lighting	0.02	4.4	0.07	12.76
	Heating	0.23	42.84	0.27	54.06
	Cooling and ventilation	0.1	16.69	0.29	48.4
Mountainous–new technology	Feeding	0.02	3.62	0.06	10.5
	Lighting	0.01	1.43	0.03	4.15
	Heating	0.28	63.14	0.27	57.27
	Cooling and ventilation	0.07	14.62	0.21	42.4
Mountainous–old technology	Feeding	0.02	0.04	2.81	8.15
	Lighting	0.01	0.02	1.02	2.97
	Heating	0.39	0.41	75.32	76.31
	Cooling and ventilation	0.06	0.15	10.22	29.64

As expected, feeding energy indices are the same for all examined cases since they are not dependent on position and technology level (even old technology level units have automated feeding systems). The same remark holds for lighting, which represents a very small percentage of energy consumption. Energy for heating decreases from mountainous old technology level units to mountainous new technology units due to thermal insulation and higher efficiency equipment. A lower energy index for cooling and ventilation is observed in mountainous old technology chambers, due to reduced needs for cooling and to the absence of relative equipment. The highest values appear in lowland old technology chambers due to increased needs for cooling and low efficiency used equipment. Cooling and ventilation represent the second bigger energy consumption in terms of final energy and the higher energy consumption in terms of primary energy in lowland units.

In conclusion, according to the above-described tables, the best behavior is achieved in the lowland new technology chambers, while the worst behavior is met in the lowland old technology chambers since they consume too much electrical energy in a non-efficient way. Mountainous chambers with old technology have a primary energy index per housing area very close to mountainous chambers with new technology. This is attributed to the fact that old technology chambers have lower electrical-powered equipment at the expense of the final product. The last is proved by the energy index per produced weight.

## Discussion

### Energy Audit Results

In order to evaluate our results, the results of other relevant works were investigated. According to a review paper (3) in 1989, 71% of the total energy consumption was used for heating, 18% was used for feed and water distribution and for manure removal, 7% was used for lighting, and only 4% was used for ventilation. In this review, an increase in energy needs in two broiler farms of 10,000 birds in Saskatchewan of Canada is reported. The annual LPG consumption for a well-insulated broiler house was 188,000 kWh and became 214,000 kWh for a poorer-insulated chamber, with the electrical annual energy consumption being 24,000 and 20,000 kWh respectively.

Later, in the 21st century, in 2007, the feasibility of an expensive renovation was examined, concluding that this depends on farm location, energy costs, and management strategy (4). According to measurements in Sweden in 2008 (5), the electricity consumption per bird was 0.13 kWh/bird. Another 0.78 kWh/bird must be added for heating and manure handling. In 2009, Liang et al. (6) measured the electrical energy consumption in a renovated chamber to 0.102 kWh/kg in Northwest Arkansas. In 2012 (7), the total energy consumption in an insulated broiler house in Finland was measured to 1.83 kWh/kg. This measurement corresponds to electrical energy consumption for lighting (0.009 kWh/kg), for ventilation (0.021 kWh/kg), and for heating (1.8 kWh/kg). It is obvious that the mechanization of broiler houses increased the percentage of electrical energy consumed for ventilation compared to the ‘80s. In 2016, in surveying concerning 29 broiler farms in Turkey, the machinery energy consumption was 0.078 kWh/bird (8).

The calculated electrical energy consumption, from our work, is of the order of 0.12–0.16 kWh/kg for lowland farms with increased cooling loads and of 0.07 kWh/kg for mountainous farms. This is in agreement with other researchers' findings' who refer consumption of 0.102 kWh/kg in Arkansas in 2009 (6) and 0.078 kWh/kg in Turkey in 2016 (8), while being at odds with predictions of 0.03 kWh/kg in Finland in 2012 (7) with characteristically low cooling demand (0.021 kWh/kg in Finland for ventilation and 0.06 to 0.1 kWh/kg for cooling and ventilation in Greece). Nevertheless, the energy consumption for lighting in the examined cases was 0.01 kWh/kg which agrees with the Finland predictions (7) of 0.009 kWh/kg. The total final energy consumption per bird varies from 0.66 kWh/bird (lowland–new technology farms) to 1.05 kWh/bird (mountainous–old technology farms), which coincides with measurements of 0.91 kWh/bird consumption in Sweden (5) at 2008, although this depends on the final birds' weight.

From the results, it is obvious that the bigger energy consumer is the heating, especially for mountainous farms. This is in line with other researchers' findings in [Michigan 1989 71% (3), Sweden 2008 85% (5), Finland 2012 98% (7)], especially in the case of mountain chambers where heating represents 84% of energy consumption. Heating is usually provided with thermal energy. However, electrical energy consumption is also important, especially in lowland farms. The biggest percentage of electrical consumption is due to ventilation and cooling (73% for the new technology farms and 67% for the old technology farms); this is also in line with the findings of (7) where they found that 70% of the electrical energy is consumed for ventilation. In electrical energy consumption, the bigger share belongs to ventilation and cooling. Feeding represents a standard consumption. Lighting energy consumption can be significantly reduced with the use of energy-efficient lights as is proved in the new technology chambers.

As far as final energy consumption in the lowland farm is concerned, new technology offers a 31% energy save compared with old technology chambers. For mountainous farms, this save is restricted to 27% which is yet important. The final energy consumption in lowland farms is 30–34% lower than in mountainous farms. The energy saving for heating due to insulation of the energy consumption is of the order of 30% higher than the 10% predicted for Canada in 1988 (3).

In terms of primary energy, new technology offers a 27% energy save in lowland farms. In mountainous farms, the primary energy reduction achieved with new technology is 24%. Since in lowland farms the share of electrical energy is big, the achieved reduction of primary energy consumption in lowland farms in comparison with the mountainous varies from 2 to 7%.

Finally, the CO_2_ emissions can be calculated from the split energy consumption presented in Table 4. Thus, lowland new technology chambers present a 26% reduction in CO_2_ emission compared to old technology. Mountainous new technology chambers reduce CO_2_ emissions by 22%. However, the emitted CO_2_ by the lowland farms is 7–11% higher than the mountainous farms' emissions.

### Proposals to Improve the Energy Performance of Broiler Facilities

#### Energy-Saving Measures

The energy consumed in broiler units for heating varies from 55% in lowland farms to 85% in mountainous farms. Heat losses in a broiler house have two basic sources. The first is the heat losses through the chamber shell due to conduction–convection. These losses are directly affected by the building insulation. The second source of loss is ventilation since the necessary fresh air that is supplied to the building must be air conditioned (e.g., heated or cooled). The reduction of these energy losses can be achieved either with precise control of the supplied fresh air either with heat recovery from the exhaust air.

According to the analysis presented in (32), an insulation thickness of 4–5 cm is appropriate for small and big chambers since thicker insulation cannot offer further significant benefits. For the mountainous chamber, a little bit thicker insulation of 6–7 cm to achieve proper insulation levels is proposed. The smaller the size of the chamber, the greater the role of insulation. A chamber without any insulation can have up to three times the thermal needs of an elementally insulated one, especially when the insulation concerns the roof. In mountainous farms, the losses through the walls are comparable to the losses due to ventilation and therefore the cost of insulation as a function of energy costs determines the optimal thickness.

When an adequate Um value has been achieved, the ventilation losses become the big source of heat losses. In an insulated lowland chamber, the ventilation heat losses are three times the shell heat losses, while in an insulated mountainous chamber the ventilation heat losses are twice the shell heat losses. In practice, this is much bigger since farmers used to supply much more than the necessary fresh air in the chambers.

Thus, the next proposed measure for energy saving is the precise control of the supplied fresh air according to the real needs of birds. For this, the existence of a net for the measurement of internal microclimate inside the chamber is necessary. These measurements contain temperature, humidity, airspeed, and NH_3_ concentration. Since the existence of such a net is expensive, the measurements can concern only a few sensors provided that software will be used to assess the real microclimate in the whole chamber and that these few sensors are located in the appropriate positions inside the chamber. A system for the precise control of ventilation also includes inverter-equipped fans controlled by a central unit.

The use of automation in heating and cooling also can offer significant energy saving as shown by the comparison of new and old technology chambers. Automation in feeding and water supply equipment is already commonplace in all types of broiler farms. Further energy consumption reduction can be achieved with the use of motors equipped with inverters.

The use of energy-efficient lights can offer energy save of the order of 5% as proved by the energy audit results. Two other general measures are the correct sizing of the energy-consuming electromechanical equipment, as there is usually a tendency to oversize and the use of electromechanical equipment with high efficiency. Finally, a general measure for energy saving is the proper maintenance of the equipment that will allow it to work at the optimum degree of efficiency.

#### Local Energy Production

Broiler units can also be energy producers. The local energy production can improve the units' energy balance, reducing the energy intensity of the breeding. In order to size RES systems, the time profile of consumption must be known.

The use of photovoltaics for local energy production is a very attractive choice for a broiler house since large roof areas are available. In fact, in a broiler house, the entire roof is available regardless of orientation due to the small angle of the pitched roof. The cost of produced kWh from PV depends on the installed power and available solar potential. Thus, in Greece, this cost ranges from 0.13 €/kWh for a small installation of the order of 3 kW to 0.073 €/kWh for an installation of 20 kW and up to 0.063 €/kWh for an installation of 100 kW. An auditor can examine three scenarios: (i) power production for sale to the grid, (ii) power production for net-metering (which is a billing mechanism that credits solar energy system owners for the electricity they add to the grid), and (iii) stand-alone PV installation with batteries for energy autonomy.

The first scenario can be examined for the cases in which the price of sale of kWh to the grid is higher than the cost of produced energy.

The second scenario, in the countries where the net-metering holds, usually is the preferred scenario since the cost of energy production by PV should be compared with the cost of purchasing the energy from the grid. In the case of net-metering, the annual energy production from PV is calculated and compared with the annual demand. The PV configuration that gives the minimum possible negative annual balance value is chosen as optimal. For the solar potential of Greece, this may lead to an installed power of (i) 8 kW for a small mountainous chamber (600 m^2^), (ii) 13 kW for a small lowland chamber (600 m^2^), (iii) 16 kW for a big mountainous chamber (1,200 m^2^), and (iv) 25 kW for a big lowland chamber (1,200 m^2^).

Finally, the cost of kWh for a stand-alone system is usually higher from 0.28 to 0.35 €/kWh according to (37). Thus, the stand-alone PV system may be attractive only for isolated units.

For a low wind potential, with a yearly average wind velocity of the order of 3.5 m/s at a height of 10 m and according to the yearly time profile of electrical power consumption, a wind turbine of (i) 10 kW for a small mountainous chamber (600 m^2^), (ii) 15 kW for a small lowland chamber (600 m^2^), (iii) 20 kW for a big mountainous chamber (1,200 m^2^), and (iv) 25 kW for a big lowland chamber (1,200 m^2^) will be needed. For such low wind potential, the chosen wind turbine is required to have a rated wind velocity of the order of 7 m/s and a cut-in wind velocity of the order of 2 m/s. However, it is not very easy to find wind turbines to cover these requirements. The cost of energy production varies from 0.18 to 0.28 €/kWh. Thus, the use of wind turbines in a low wind potential could be attractive only if the cost of purchase of electricity from the grid is higher or is in isolated areas. However, if the wind potential is important the cost of energy production may decrease to 0.05 €/kWh.

Solar thermal energy can be used to cover the self-consumption for biogas production. Since initial heating of the biogas reactor requires high temperatures that must be achieved in a short time, it will be considered that these will be covered by burning biogas and only the heat losses of the reactors will be covered by thermal solar systems. Underfloor heating may be considered only for new chambers. Nevertheless, this requires important modification to the chamber basic construction since it requires replacement of the bedding with flooring with special specifications that allow the birds to live safely, have special consideration for manure management, and do not impede heat transfer. Another way to utilize thermal solar energy is in combination with heat pumps provided that the appropriate air duct heating system has been selected. Since there is no need for a high water temperature in the above applications, the proposed type is the flat selective collector. The use of concentrating solar collectors in these applications would not offer an advantage.

Shallow geothermal systems combined with (38) both heat pumps and soil heating applications to agricultural activities (e.g., asparagus) proved advantageous, resulting in a discounted thermal energy unit cost of <45 €/MWhth contributing an internal rate of return on investment up to 24%. Nevertheless, in existing poultry facilities, the use of shallow geothermal energy would require the use of an underfloor heating system or the collaboration with a heat pump. The cost of replacement of existing heating/cooling systems only for improving the energy efficiency is considered prohibitive.

The basic method that is suggested for the utilization of the produced biomass of broiler farms is anaerobic fermentation (39–41). The raw material used as biomass is bird manure mixed with the litter since in this type of unit no separation can be done. According to the literature, anaerobic fermentation leads to biogas production (with 50–60% CH_4_). This method is well established in some livestock facilities (e.g., pigsties, cowsheds); however, in the case of broiler farms, some particular problems are faced in the application of this method: (i) discontinuous feeding of the reactor with biomass, (ii) requirement to purchase necessary additives (to set required C:N ratio), (iii) water management, (iv) self-consumption for the reactor operation, and (v) energy utilization of discontinuously produced biogas (the biogas will be produced when the breading is over and so it should be stored).

Unlike in other livestock facilities where manure is collected on a daily basis or at a fixed time step and has a constant supply over time, in broiler farms manure can only be collected at the end of the breading (five times a year).

If the bedding is straw before feeding it to in the reactor, pretreatment should be done to reduce the size of the straw pieces. Regardless of the type of litter (straw or rice husks) before the introduction of the mixture into the reactor, additional material from agricultural residues should be added for the mixture to obtain the necessary organic load (set to required C:N ratio 20–40). Of course, the amount and characteristics of the additives depend on the type of litter since a different litter means a different chemical composition of the collected biomass.

In any case, water should be added if the humidity of the specific livestock waste is very low (required dry matter for the case of horizontal reactor 15–20%, while for the case of vertical reactor 10–15%). The management of the water used becomes a major problem since it must be cleaned of nitrogen before being reused or disposed of in the environment.

The produced biogas must be purified from H_2_S before its use. The biogas can be used either to generate electricity by supplying an internal combustion engine that drives an electric generator, or to generate heat by combustion in a gas boiler, or to simultaneously generate electricity and heat in a combined heat and power (CHP) unit.

Here are some general guidelines for the technology used. The use of a vertical reactor (300–1,500 m^3^) with a batch operation is suggested. Initial heating for 1 h at 70°C and fermentation in the mesophilic area (35°C), with residence time in the reactor, is 35–40 days.

No further general guidelines can be given, and each case should be studied individually according to the size of the chambers, the type of used litter, the type of used heating, and cooling systems and mainly the timing of breeding between the different chambers of a unit. In a multichamber broiler farm, proper synchronization of breeding between chambers can reduce the problem of discontinuous biomass production. In addition, in the case where several units use a common biogas unit, the same can be achieved by synchronizing the breeding between the different farms, provided, of course, that the timing of the breeding does not affect other more important parameters of the breeding.

## Conclusions

The consumed energy at poultry facilities varies from 46 to 89 kWh/m^2^ of chamber area or from 0.25 to 0.48 kWh/kg of produced meat depending on the chamber technology level (insulation, automation, etc.) and the location where the unit is installed. However, in terms of primary energy, the above energy indices become 91–126 kWh/m^2^ and 0.5–0.69 kWh/kg. The bigger energy consumer is heating followed by energy consumption for ventilation and cooling. Advanced technology levels can improve energy performance up to 27–31%.

Proper insulation (4–7 cm depending on the location) can offer a reduction of thermal energy consumption between 10 and 35%. In adequately insulated chambers, the basic heat losses are due to ventilation. Thus, further energy saving can be achieved with precise control of ventilation according to the real needs of birds. The use of automation can offer an additional save of electrical energy consumption for cooling and ventilation (15–20%). The use of energy-efficient lights can offer energy savings up to 5%.

Energy intensity in broiler facilities can be reduced through local energy production. The use of PV is suggested mainly in areas where net-metering holds. The use of wind turbines is feasible only when adequate wind potential is available to reduce the cost of producing energy lower than the cost of purchasing energy from the grid or for isolated areas. A thermal solar system is suggested in combination with a heat pump if adequate systems for heating and cooling are used.

Finally, the local production of biogas with anaerobic fermentation for producing thermal or electrical energy, or cogenerating both, is a choice that should be studied individually for each farm depending on the type of the litter, the synchronization among the breading of different farms, and the availability of additives. In any case, special attention must be paid to the management of the water that will be used to add to the biomass for the necessary moisture.

The presented energy audit protocol can be a useful tool to reduce the energy and environmental footprint of broiler farms.

## Data Availability Statement

The original contributions presented in the study are included in the article/supplementary material, further inquiries can be directed to the corresponding author.

## Author Contributions

CB and DF: conceptualization, methodology and writing–original draft preparation. CB, DF, IG, EB, and IS: validation, investigation, data curation, writing–overview, and editing. IS: funding acquisition. All authors have read and agreed to the published version of the manuscript.

## Conflict of Interest

The authors declare that the research was conducted in the absence of any commercial or financial relationships that could be construed as a potential conflict of interest.

## Publisher's Note

All claims expressed in this article are solely those of the authors and do not necessarily represent those of their affiliated organizations, or those of the publisher, the editors and the reviewers. Any product that may be evaluated in this article, or claim that may be made by its manufacturer, is not guaranteed or endorsed by the publisher.

## Abbreviations

PV: photovoltaic
GHGs: greenhouse gas emissions
RES: renewable energy sources
PV/T: photovoltaic/thermal
EM: electromechanical
HDD: heating degree days
CDH: cooling degree hours
CHP: combined heat and power.
